# Risk factors for *Toxoplasma gondii* seropositivity in the Old Order Amish

**DOI:** 10.1017/S0950268820002897

**Published:** 2020-11-25

**Authors:** A. O. Markon, K. A. Ryan, A. Wadhawan, M. Pavlovich, M. W. Groer, C. Punzalan, K. Gensheimer, J. L. Jones, M. L. Daue, A. Dagdag, P. Donnelly, X. Peng, T. I. Pollin, B. D. Mitchell, T. T. Postolache

**Affiliations:** 1 Division of Public Health Informatics and Analytics (DPHIA), U.S. Food and Drug Administration, Center for Food Safety and Applied Nutrition (CFSAN), Office of Analytics and Outreach (OAO), College Park, MD, USA; 2 Division of Endocrinology, Diabetes and Nutrition, Department of Medicine, University of Maryland School of Medicine, Baltimore, MD, USA; 3 Program for Personalized and Genomic Medicine, University of Maryland School of Medicine, Baltimore, MD, USA; 4 Department of Psychiatry, Mood and Anxiety Program, University of Maryland School of Medicine, Baltimore, MD, USA; 5 Department of Psychiatry, Saint Elizabeth’s Hospital, Washington, DC, USA; 6 College of Nursing, University of South Florida College of Nursing, Tampa, FL, USA; 7 Geriatrics Research and Education Clinical Center, Veterans Affairs Medical Center, Baltimore, MD, USA; 8 Amish Research Clinic, University of Maryland School of Medicine, Lancaster, PA, USA; 9 Rocky Mountain Mental Illness Research Education and Clinical Center (MIRECC), Veterans Integrated Service Network (VISN) 19, Military and Veteran Microbiome: Consortium for Research and Education (MVM-CoRE), Denver, CO, USA; 10 Mental Illness Research, Education and Clinical Center (MIRECC), Veterans Integrated Service Network (VISN) 5, VA Capitol Health Care Network, Baltimore, MD, USA

**Keywords:** Amish, food safety, *Toxoplasma gondii*

## Abstract

*Toxoplasma gondii (T. gondii)* is an important human disease-causing parasite. In the USA, *T. gondii* infects >10% of the population, accrues economic losses of US$3.6 billion/year, and ranks as the second leading culprit of foodborne illness-related fatalities. We assessed toxoplasmosis risk among the Old Order Amish, a mostly homogenous population with a high prevalence of *T. gondii* seropositivity, using a questionnaire focusing on food consumption/preparation behaviours and environmental risk factors. Analyses were conducted using multiple logistic regression. Consuming raw meat, rare meat, or unpasteurised cow or goat milk products was associated with increased odds of seropositivity (unadjusted Odds Ratios: 2.192, 1.613, and 1.718 , respectively). In separate models by sex, consuming raw meat, or consuming unpasteurised cow or goat milk products, was associated with increased odds of seropositivity among women; washing hands after touching meat with decreased odds of seropositivity among women (adjusted OR (AOR): 0.462); and cleaning cat litterbox with increased odds of seropositivity among men (AOR: 5.241). This is the first study to assess associations between behavioural and environmental risk factors and *T. gondii* seropositivity in a US population with high seroprevalence for *T. gondii*. Our study emphasises the importance of proper food safety behaviours to avoid the risk of infection.

## Introduction

The apicomplexan parasite *Toxoplasma gondii* (*T. gondii*) infects one-third of the world's population, disproportionately affects socio-economically disadvantaged groups, and ranks among five neglected parasitic infections targeted in the USA by the Centers for Disease Control (CDC) for public health action [1, 2]. Some estimates indicate that in the USA, ~170 000 new toxoplasmosis cases occur annually and that 1.1 million are currently infected, amounting to infection among 13.2% (age-adjusted: 12.4%) of the population > 6 years of age [3]. Other sources note that although >40 million men, women and children in the USA may harbour the parasite without symptoms because the immune system prevents the occurrence of the full-blown illness, consequences of infection can be severe for the immunocompromised and for women infected during or shortly before pregnancy [2].

The burden of *T. gondii* infection by foodborne routes, which is estimated to account for about half of all US cases, exceeds $3.6 billion and equates to the loss of 459 481 quality-adjusted life days [4]. According to Scallan *et al*. (2011), 2.6% of US-acquired foodborne *T. gondii* infections will result in hospitalisations, and 0.02% will result in death, leading to an estimated 327 deaths annually, making toxoplasmosis the second leading cause of foodborne illness fatality in the USA [5].

**Fig. 1.**
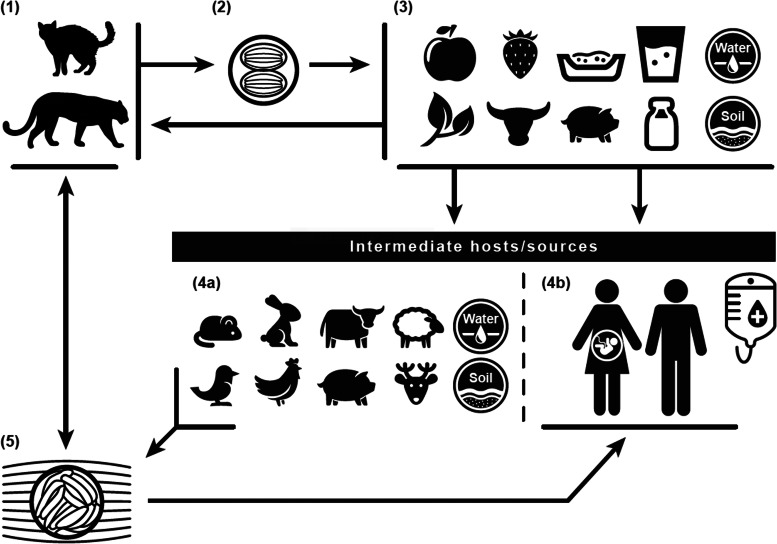
Toxoplasma gondii life cycle: The definitive felid hosts, including house cats (1), uptake the Toxoplasma gondii parasite through ingestion of tissue oocysts (5) from infected intermediates, such as rodent and bird prey, or other sources, including water (4a). Following uptake, the definitive felid hosts shed unsporulated oocysts for up to 3 weeks through feces (2), contaminating water, soil, kitty litter, and food sources (3) of intermediate hosts/sources (4a/4b). The unsporulated oocysts distributed through the definitive felid host feces take 1 to 5 days to become infective. Intermediates include the definitive felid host prey, human food sources, water, and soil (4b). Humans can contract infection through intake of contaminated food or water; contact with contaminated soil or kitty litter; blood transfusions or organ transplants; or maternal to fetal (vertical/placental) transmission.

Sexual reproduction of *T. gondii* occurs in its definitive felid hosts, including cats, while asexual reproduction occurs in intermediate hosts, including humans and nearly all warm-blooded vertebrates. The parasite has three infectious stages [6–15]: sporulated oocysts that contain sporozoites, tachyzoites that invade and multiply rapidly within non-intestinal epithelial cells of the definitive hosts and in any cell of intermediate hosts , and bradyzoites that slowly replicate in tissue cysts (life cycle detailed in Fig. 1). Tissue cysts are most often found in the central nervous system; the eyes; and smooth, skeletal or cardiac muscles. While bradyzoites in tissue cysts characterise chronic infection, tachyzoites characterise primary acute or reactivated infections, which can trigger adaptive IgG immune responses [7, 8]. In reactivation, tissue cysts rupture and release bradyzoites that transform back into destructive tachyzoites [11, 13]. Toxoplasmosis may present in immunocompetent individuals with painless lymphadenopathy; mononucleosis-like symptoms, including fever, malaise, sore throat and maculopapular rash; ocular infections in some older populations; or no symptoms in pregnant women [9, 12]. Severe clinical manifestations, such as pneumonitis, chorioretinitis or multi-organ involvement, occur most often in reactivated and primary infections among immunocompromised individuals and in congenital infections [9, 10]. HIV-positive individuals often present with life-threatening toxoplasmic encephalitis, characterised by headache, confusion, weakness, focal neurologic involvement and seizures [11, 12]. Previously, chronic infections were not associated with clinical manifestations [9], but recent studies have linked chronic infection to schizophrenia [13–15] and suicidal behaviour [16, 17].

Multiple behavioural, socio-economic and demographic factors may contribute to the risk of toxoplasmosis. Studies focusing on behavioural risk factors often use a combination of immunoassays to determine infection and questionnaires to conduct an epidemiologic assessment of potential exposures. While the questionnaires themselves are often tailored to specific populations, many ask similar questions regarding behaviour-related exposures: most notably, eating different meats [18–22] (porcine, bovine, lamb/mutton, other); handling or eating raw or undercooked meat [18, 22]; consuming dairy/dairy products [22–24]; eating unwashed or improperly washed fruits and/or vegetables [22, 25, 26]; gardening and/or engaging in contact with soil [18, 21, 22, 26]; living with pets, such as cats and/or dogs at home [18, 19, 26]; drinking water (treatment, source, other characteristics) [19, 22, 27]; and even hunting/consuming wild game (venison, boar, other) [18, 27]. In the USA, those below the federal poverty threshold have higher *T. gondii* seropositivity [3], while higher early-life socio-economic status has been shown to be related to seronegativity [28]. Other risk factors for higher seroprevalence, specifically in the USA, include foreign country of birth, Hispanic and non-Hispanic Black race/ethnicity, lower than high school education levels, crowded housing conditions, rurality/urbanicity and employment type [3, 22].

The Old Order Amish (OOA) population in the USA, by virtue of its homogeneous characteristics, provides a unique opportunity to assess relationships between *T. gondii* seropositivity and specific behavioural and environmental (including food-related) risk factors. *T. gondii* seropositivity prevalence levels among the OOA exceed those found among the general US population [29], and the geographic, ethnic and socio-economic homogeneity of the OOA population [30] helps to control the potential confounding that can otherwise complicate risk factor assessment. In addition, the Amish lifestyle presents the prospect of evaluating potential associations between *T. gondii* seropositivity and culturally specific meal-preparation practices, such as slaughtering, smoking, curing and canning meats [31]. These distinctive characteristics, in conjunction with antibody testing and data collection using OOA lifestyle-specific questions, allowed this study to pursue the objective of advancing understanding of relationships between the prevalence of *T. gondii* seropositivity and behavioural and environmental (including food-related) risk factors of infection, while minimizing confounding by other potential socio-demographic and behavioural determinants.

## Methods

### Study population

#### The Amish Wellness Study

The Amish Wellness Study (AWS), an ongoing community-based investigation conducted by the University of Maryland Baltimore and approved by the University of Maryland Baltimore Institutional Review Board, was established in 2010 to offer health screening and collection of DNA, blood samples and basic medical information to address specific research questions. Criteria for participation included membership in the OOA community of Lancaster County, Pennsylvania; age of ⩾18 years; and informed consent. A registered nurse obtained multiple clinical measures of general health (e.g., weight, height, waist and hip circumference, blood pressure, measures of respiratory health). An Amish liaison facilitated administration of the study questionnaire in person to assess several aspects of each participant's health and well-being, including mood, sleep, and medical, personal and family histories; as well as to evaluate lifestyle factors. A University of Maryland Amish Research Clinic research nurse confirmed completion of each questionnaire. A fasting blood sample was drawn for the measurement of lipids and glucose, and from this sample, we also measured *T. gondii* IgG antibodies for this study.

For the present study, we sent the Amish *Toxoplasma* Risk Factor Questionnaire (ATRFQ) to a convenience sample from the AWS (*n* = 3551, non-randomly selected, based on availability to participate), between 30 December 2015 and 3 January 2016. We evaluated participant responses to the ATRFQ, along with their demographic information and *T. gondii* antibody results from the AWS.

### Immunoglobulin G serointensity and seropositivity testing

For this study, the laboratory of Maureen Groer at the University of South Florida in Tampa determined *T. gondii* antibody seroprevalence using the enzyme-linked immunosorbent assay (IBL International, Männedorf, Switzerland) that tests for RH factor common to all *T. gondii* serotypes. Concentrations of ⩾12 IU/ml were defined as seropositive, and of 8–12 IU/m as equivocal. The equivocal samples were tested a second time, and if the second testing yielded samples that tested positive for concentrations of ⩾12 IU/ml on the second test, they were also considered positive as previously reported [32].

### Amish *Toxoplasma* Infection Risk Factor Questionnaire

The ATRFQ was developed by researchers at the University of Maryland Baltimore with input from epidemiologists at FDA's Center for Food Safety and Applied Nutrition (CFSAN) and co-authors based on current risk factor literature and subject matter expertise. The questionnaire was adapted after incorporating input from three sessions of review and feedback by a group of Amish liaisons, three sessions of feedback by nurses experienced in working with the Amish, and individual researcher comments and questions to maximise comprehension and cultural sensitivity. The questionnaire consisted of 30 questions focusing primarily on food consumption and preparation, as well as other environmental risk factors, such as water sources, pet ownership and contact, and contact with soil. The pet component specifically asked about cat/kitten ownership, cat breeding, cat litter cleaning, where the cats live (outdoors *vs.* indoors), where the cats/kittens eat, type of food they eat, visits of not-owned cats, type of cats seen in the garden or yard, cats for breeding, feral cats (domesticated and returned to the wild), and stray cats (lost or abandoned). It was mailed to study participants between 30 December 2015 and 3 January 2016 with a cover letter, response envelope and US$2 compensation. The cover letter informed participants that they could keep the $2 even if they decided not to participate. Responses from the returned questionnaire received by 11 April 2016 were entered into an Access database. Returned envelopes received after 11 April 2016 were not included in the analytical sample used for this study.

### Statistical analyses

The analytical sample included those with both *T. gondii* serological measures and ATRFQ responses. Analyses were performed to describe the analytical sample (Table 1) and to assess the strength and significance of associations with examined behavioural and environmental (including food-related) risk factors through models estimating the odds of *T. gondii* seropositivity (Tables 2–4). In addition to age and sex derived from the parent AWS survey, the ATRFQ variables addressed contact with soil, pets (including cats and litter boxes) and shoes/feet before touching food; farm *vs.* other-than-farm residence and time at residence; drinking untreated or treated water and drinking-water source/filtration; working with animals/raw meat/horses; consuming locally-produced, cooked, cured, frozen, canned, raw and/or rare meats; consuming raw oysters; consuming unpasteurised goat and/or cow milk/products; washing produce; washing hands/utensils after raw meat contact; and eating outside the home.

**Table 1.** Demographic characteristics of Old Order Amish participants, *Toxoplasma gondii* serostatus and risk factor study, 2015–2016

Characteristic	Total *n* (%) 843 (100%)	Seropositive *n* (%) 478 (56.7)	Seronegative *n* (%) 365 (43.3)	*P* value^a^
Age (years)				
18–29	180 (21.4)	77 (16.1)	103 (28.2)	*<0*.*0001*
30–39	145 (17.2)	77 (16.1)	68 (18.6)	
40–49	153 (18.2)	90 (18.8)	63 (17.3)	
50–59	138 (16.4)	75 (15.7)	63 (17.3)	
60–69	136 (16.1)	92 (19.3)	44 (12.1)	
70+	91 (10.8)	67 (14.0)	24 (6.6)	
Mean, Median, (s.d.)	46.6/45.0 (16.8)	49.3/49.0 (16.7)	43.2/41.0 (16.3)	*<0*.*0001*
Sex				
Female	507 (60.1)	262 (54.8)	245 (67.1)	*<0*.*0003*
Male	336 (39.9)	216 (45.2)	120 (32.9)	
Body mass index				
Mean (s.d.)	26.5 (4.9)	26.7 (5.0)	26.3 (4.8)	0.24
*T. gondii* titre concentration (IU/ml)^b^				
Median (s.d.)	15.6 (3.4, 77.2)	67.9 (24.6, 113.6)	2.8 (1.5, 4.9)	*<0*.*0001*

s.d., Standard deviation; *T. gondii*, *Toxoplasma gondii*.

a *P* value from *χ*^2^ test of independence for age and sex [33]; from SAS 9.4 for *t* test; italics denote significance at *P* < 0.05.

b *T. gondii* titre concentration: seropositive ⩾12 IU/ml; seronegative <12 IU/ml.

**Table 2.** Bivariate analysis of unadjusted associations (unadjusted odds ratios) of serostatus and category-based behavioural risk and protective factors of Old Order Amish participants, *Toxoplasma gondii* serostatus and risk factor study, 2015–2016

Behavioural risk or protective factor		Seropositive of those positive for behaviour *n*/*n* (%)	Seropositive of those negative for behaviour *n*/*n* (%)	Unadjusted odds ratio	95% CI	*P* value
Pets (ref: no pets)	Has pets	340/622 (54.7)	127/204 (62.3)	0.959	0.670–1.372	0.818
	Has cats	267/455 (58.7)	211/388 (54.4)	1.209	0.912–1.603	0.187
	Has kittens	164/287 (57.1)	314/556 (56.5)	1.096	0.811–1.481	0.549
	Has contact with cats	364/638 (51.1)	114/205 (55.6)	1.134	0.818–1.571	0.452
	Cleans the litterbox	30/40 (75.0)	396/703 (56.3)	2.451	1.167–5.143	*0*.*018*
Meat consumption (ref: no consumption)	Ate rare meat	62/95 (65.3)	416/748 (55.6)	1.613	1.017–2.560	*0*.*042*
	Ate raw meat	57/96 (59.4)	421/747 (56.4)	2.192	1.172–4.101	*0*.*014*
	Ate raw ground pork^a^	1/1 (100.0)	471/836 (56.3)	∞	∞	*<0.05*^a^
	Ate rare ground beef	19/25 (0.76)	459/818 (56.1)	2.568	0.996–6.623	0.051
Unpasteurised milk consumption^b^ (ref: no consumption)	Consumed unpasteurised cow or goat milk products	423/735 (57.6)	55/108 (50.9)	1.718	1.116–2.643	*0*.*01*
Washing hands (ref: not always washing hands after touching raw meat)	Always washing hands after touching raw meat	348/636 (54.7)	80/125 (64.0)	0.642	0.426–0.950	*0*.*035*

Italics denote significance at *P* < 0.05.

a Only one respondent indicated raw ground pork intake.

b Only two respondents indicated consumption of unpasteurised goat milk products; 421 indicated intake of unpasteurised cow milk products.

**Table 3.** Bivariate analysis of associations (unadjusted odds ratios) of serostatus and frequency-based behavioural risk and protective factors of Old Order Amish participants, *Toxoplasma gondii* serostatus and risk factor study, 2015–2016

Behavioural risk or protective factor		Never	<1/week	⩾1 week	Daily	Unadjusted odds ratio	95% CI	*P* value
Consumption of	Unpasteurised cow milk/yoghurt	57/112 (50.9)	47/82 (64.0)	77/131 (58.8)	297/ 518 (57.3)	1.211	1.055–1.391	*0*.*007*
	Rare meat	416/748 (55.6)	14/20 (70.0)	2/3 (66.7)	1/1 (100.0)	1.434	1.023–2.011	*0*.*037*
	Raw meat	440/790 (55.7)	2/3 (66.7)	1/1 (100.)	0/0 (n/a)	2.003	1.120–3.584	*0*.*019*
		Less than once a month		Once a month or more		Unadjusted odds ratio	95% CI	*P* value
Location of meal consumption	Outside the home	334/570 (58.6)		75/148 (50.7)		0.611	0.418–0.984	*0*.*01*

Italics denote significance at *P* < 0.05.

**Table 4.** Adjusted associations (adjusted odds ratios) of *Toxoplasma gondii* seropositivity and age and behavioural risk and protective factors by sex of Old Order Amish participants, *Toxoplasma gondii* serostatus and risk factor study, 2015–2016

		Male			Female		
Factor		Adjusted odds ratio	95% CI	*P* value	Adjusted odds ratio	95% CI	*P* value
Age	Female				1.027	1.016–1.038	*<0*.*0001*
	Male	1.015	1.001–1.030	*0*.*0348*			
Pets	Has pets	0.819	0.442–1.520	0.5273	1.048	0.673–1.632	0.8364
	Has kittens	1.408	0.882–2.247	0.1511	0.917	0.617–1.363	0.6684
	Has cats	1.112	0.704–1.758	0.6481	1.278	0.892–1.830	0.1814
	Cleaned the litterbox	5.241	1.165–23.584	*0*.*0309*	1.466	0.578–3.723	0.4205
Meat consumption^a^	Ate rare meat	1.765	0.901–3.458	0.0978	1.45	0.762–2.760	0.2579
	Ate raw meat	1.111	0.446–2.77	0.8216	3.536	1.469–8.510	*0*.*0048*
	Ate rare ground beef	2.988	0.640–13.96	0.164	2.327	0.687–7.889	0.175
Unpasteurised milk consumption^b^	Consumed unpasteurised cow or goat products^b^	1.481	0.719–3.050	0.2872	1.863	1.086–3.197	*0*.*0239*
	Consumed unpasteurised cow milk products	1.450	0.719–2.926	0.2996	1.930	1.127–3.304	*0*.*0166*
Washing hands	Always washing hands after touching raw meat	0.931	0.519–1.672	0.8115	0.462	0.26–0.823	*0*.*0087*

Italics denote significance at *P* < 0.05.

a Only one respondent indicated raw ground pork intake; results, therefore, not shown.

b Only two respondents indicated consumption of unpasteurised goat milk products; 421 indicated intake of unpasteurised cow milk products.

Bivariate analyses, including *χ*^2^ and *t* tests, compared seropositive to seronegative to identify significant or borderline significant (*P* value <0.05 and <0.20) variables. For those variables, we then specified comparison reference groups and estimated unadjusted odds ratios (UORs), 95% confidence intervals (CIs) and *P* values (Tables 2 and 3). We included the variables that exhibited significance in the bivariate UOR analysis (*P* value <0.05) in the final unconditional multiple logistic regression models by sex to estimate adjusted odds ratios, 95% CIs and *P* values, evaluating the associations between each of the predictor variables and the outcome of *T. gondii* seropositivity as defined above (Table 4). These analyses were performed using SAS 9.4 (Cary, NC, USA), as well as the *χ*^2^ calculator in McDonald (2014) [33]. We also adjusted the results for relatedness (heritability) and shared households by applying a variance component approach using SOLAR 6.6.2 software (San Antonio, TX, USA) to assess significance (*P* value <0.05) [34].

## Results

Of the 3551 questionnaires mailed to study participants, 1518 (43%) were returned. *Toxoplasma gondii* serology was assessed in 55.5% (*n* = 843) of those who returned questionnaires. We limited our analytical sample to the 843 Amish subjects with both serology test results and questionnaire response data (23.7% of the 3551 who were mailed the questionnaire also had serology results).

More than half (56.7%) of the 843 study participants tested positive for *T. gondii* IgG antibodies (Table 1). Nearly two-thirds of participants (60.1%) were female among both the seropositive and seronegative groups. The age distribution/*n* was similar among seropositive individuals, but not among seronegative individuals or the whole study sample. Mean and median ages were higher among seropositive than among seronegative individuals, and there were more seropositive than seronegative males.

In addition to sex, the *χ*^2^ test results from the first step of the bivariate analysis indicated significant/borderline significant associations between *T. gondii* seropositivity (*P* values <0.20) and the category-based variables capturing farm *vs.* other-than-farm residence and time at residence; working with horses; consuming unpasteurised cow milk/products; consuming cured, rare and raw meats; pet contact; and washing hands/utensils after raw meat contact, as well as the frequency-based variables addressing how often unpasteurised cow milk/products, raw meats, rare meats and meals outside the home were consumed.

Of those variables, the UOR estimates from the second step of the bivariate analysis showed significance (*P* value <0.05) for pet contact, consumption of meat and unpasteurised milk/products, and handwashing behaviours (Table 2), as well as for the frequency of consumption of unpasteurised cow milk/yoghurt, rare meat, raw meat and meals outside the home (Table 3).

The meat-eating behaviours, as well as the two variables addressing the frequency of consumption, were all associated with *T. gondii* seropositivity. The odds of seropositivity from eating rare and raw meat compared to not eating rare and raw meat were 1.613 (95% CI 1.017–2.560) and 2.192 (95% CI 1.172–4.101), respectively. Only one individual reported eating raw ground pork and was seropositive. Odds of seropositivity comparing the consumption of unpasteurised milk to not consuming raw milk was 1.211 (OR 1.211, 95% CI 1.055–1.391). Among the behaviours related to pets, only cleaning a cat's litterbox was found to be statistically significantly associated with *T. gondii* seropositivity (OR 2.451, 95% CI 1.167–5.143).

Because of the significant association found between sex and seropositivity in the bivariate analysis, separate models by sex were used to estimate the adjusted associations between the other variables also identified as significantly associated with seropositivity (Table 4). Several of the associations between *T. gondii* seropositivity and behavioural and environmental (including food-related) risk factors did not retain significance in the adjusted models by sex. However, the separate adjusted models by sex showed that significance persisted nominally for cleaning the litterbox (among men only), eating raw meat (among women only) and washing hands after touching raw meat (protective in women only). Consuming raw goat or cow products, as well as unpasturised cow milk products, weresignificantly associated with seropositivity among women only (OR 1.863, 95% CI 1.086–3.197; OR 1.930, 95% CI 1.127–3.304, respectively, where only two respondents indicated consumption of unpasteurised goat milk products, and 421 indicated intake of unpasteurised cow milk products). Washing *vs.* not washing hands after touching raw meat was significantly negatively associated with seropositivity among women only (OR 0.462, 95% CI 0.260–0.823). These adjusted significant results were held, even after adjustment for heritability and shared household.

## Discussion

This study found that members of the OOA community of Lancaster, Pennsylvania have a high seroprevalence of *T. gondii* antibodies, with over 56% of study participants testing seropositive, exceeding the levels reported in many other US populations, although similar to levels reported in Java, Indonesia (62.5%) [24] and among the Nunavik Inuit of Canada (59.8%) [27]. The Jones *et al*.'s (2014) US-based analysis of the 2009–2010 National Health and Nutrition Examination Survey (NHANES) that included a wave of participants aged ⩾6 years found a much lower seroprevalence of 13.2% (95% confidence limit (CL) 11.8–14.5%) prior to adjustment for age and an age-adjusted seroprevalence of 12.4% (95% CL 11.1–13.7%) [3]. As these studies vary in terms of recruitment and analytical methodologies, we suggest caution when interpreting these differences. The uniqueness of our study population may further complicate comparisons – as previously mentioned, the OOA community is an ethnically, behaviourally and culturally distinct group. We are not aware of any studies assessing the seroprevalence of *T. gondii* in other Amish populations and have found only one study focusing on a similar population. Alvarado-Esquivel *et al*. (2010) conducted a *T. gondii* risk factor and seroprevalence analysis among 152 Mennonites in Durango, Mexico, an isolated and mostly rural population of ethnic Germanic descent, with cultural and behavioural aspects that differ from the surrounding communities [35]. They found lower seroprevalence (30.3%) among the Mennonites of Durango [35], compared to the 56% seroprevalence rate that we have observed in the OOA community of Lancaster, Pennsylvania. Also, the seroprevalence of *T. gondii* IgG antibodies in the general population of Durango City, Mexico was reported to be 6.1%, which was remarkably less than the seroprevalence in the Mennonite community living in the same city [36]. This difference in seroprevalence may relate to diverse factors, including food sources, diet, unique group practices, geographic characteristics and other factors that differ between this Mennonite community and the OOA.

The rural location of residence may contribute to the higher seroprevalence among the OOA compared to other populations. Seroprevalence studies often consider living in a rural environment a risk factor because of the presence in rural settings of potentially infected animals that can transmit the parasite to humans in a variety of ways [12]. The finding of higher seroprevalence among rural compared to urban populations is consistent with the results from the UK, Germany and Ireland [21, 37, 38]. In addition, a 2013 study by Muñoz-Zanzi *et al*. found that children residing on farms in Wisconsin were more likely to be seropositive than those who did not reside on farms [39]. In their multivariate analysis of the 1988–94 wave of NHANES, Jones *et al*. (2001) reported significantly lower seropositivity among non-Hispanic Blacks living in metropolitan areas (>1 million population) (OR 0.74, 95% CI 0.59–0.93) than among non-Hispanic Blacks living in non-metropolitan areas [40]. Yet, the risk of *T. gondii* infection was not different among residents of metropolitan and non-metropolitan areas for populations of non-Hispanic Whites or Mexican-Americans [40]. Our study population lacked a proper urban counterpart to assess the impact of rurality/urbanicity, so we cannot exclude the possibility that there is simply high seroprevalence in the general study area.

Several of the food- and food safety-related risk factors found to be significantly associated with *T. gondii* seropositivity in this study are consistent with results from other studies. First, consumption and handling of raw and/or undercooked meats was associated with *T. gondii* seropositivity for both beef and pork, as previously seen in the literature [18, 21–26]. While the practice of cleaning a cat's litterbox was also observed to be a risk factor among males in our study, our findings highlight the importance of the foodborne transmission route for *T. gondii*. In addition, washing hands after handling raw meat was found protective of *T. gondii* seropositivity in our study, especially among women. Our findings emphasise the importance of proper food safety behaviours, such as cooking meat to a safe temperature prior to eating/not consuming raw meat, which is effective at diminishing the risk of contracting the parasite. Also, people should avoid accidental contamination by thoroughly washing hands before and after handling meats, and properly washing preparation surfaces and utensils (such as knives) with soap and hot water after use. Washing of hands and/or use of gloves during gardening may also help prevent contamination [2]. Our study also found that consumption of unpasteurised milk products was associated with the risk of seropositivity. While intake of these products provides a possible route of exposure to the parasite [22–24], consumption rates are low among the US population [22, 41, 42], and thus could explain, in part, the increased seropositivity among the Amish.

This study provided novel insights into risk factors for infection with a highly prevalent parasite in a unique US population. Among several strengths, the study population is known for its ethnic and socio-economic homogeneity, which may have helped to minimise confounding by unmeasured variables. Second, response rates were high for both the AWS questionnaire and the ATRFQ. Third, to counteract the possibility that the participants' close family and household aggregation could have produced spurious associations, the study adjusted post-hoc for heritability and household [32], without any loss of statistical significance for any of the positive findings reported in our study.

Among the study's important limitations, the findings may not be generalisable to other populations in the USA or elsewhere, given the unique characteristics of the culturally distinct OOA [30]. In addition, we saw different distributions between age groups, which may be due to cumulative seropositivity and may have biased some of the observed associations. Our study also included only adults (age 18 years or older), which means that we are unable to assess whether infections were acquired during childhood. Consequently, we are unable to access certain risk factors and are also unable to provide recommendations to prevent these potential exposures. This study is also limited by a relatively small sample size given the number of risk factors analysed. Although the high seropositivity in the OOA may have mitigated sample size issues, the low incidence of certain behaviours may have masked the statistical significance of their potential association with seropositivity.

We presented our findings, both formally and informally, to the OOA community in Lancaster, PA. We discussed the different *T. gondii* risk factors identified in our analysis and specifically addressed the observed sex-based differences. Direct feedback from the OOA community and the findings themselves have and will continue to be used to develop content for future education efforts, including food safety. We plan on applying for grants to develop additional educational materials, train Amish educators and conduct future studies to assess the possible relationships between knowledge and practice among the OAA. For example, future research efforts include developing a study to assess *Toxoplasma* risk factors and foetal infection, morbidity and mortality, and possibly establishing a longitudinal study to look at a variety of other important genetic, epigenetic, proteomic, clinical and environmental factors, with sampling data for *T. gondii* in food, soil, water, and farm and domestic animals, as well as neuroimaging and other markers of physical and neuropsychiatric health and functioning.

## Conclusion

This study found *T. gondii* seropositivity among more than half of the participants from a unique OOA community with culturally specific meal preparation practices. The study highlights significant behavioural and environmental (including food-related) risk factors associated with infection, such as consuming undercooked meats and unpasteurised cow milk products, as well as protective behaviours, such as eating meals outside of the home and washing hands after contact with raw meat, showing the importance of proper food safety practices for meal preparation, which is of growing relevance to the consumers in the current general US population as well. Future research will focus on characterizing specific microorganism traits, including serotypes, markers of infection, and genetic, physiological and clinical associations with *T. gondii* seropositivity, as well as advancing the understanding of education and outreach needed to promote protective behaviours and reduce the burden of toxoplasmosis from foodborne and other routes.

## Data

The data cannot be shared because of the original protocol and Amish consent. A collaborative effort could be considered and interested individuals should contact the corresponding author.

## References

[1] Jones JL, Parise ME and Fiore AE (2014) Neglected parasitic infections in the United States: toxoplasmosis. American Journal of Tropical Medicine & Hygiene 90, 794–799. 10.4269/ajtmh.13-0722 PMC4015566 24808246

[2] Centers for Disease Control and Prevention (CDC). Parasites – toxoplasmosis (Toxoplasma infection). Available at https://www.cdc.gov/parasites/toxoplasmosis/index.html. Accessed June 3, 2019.

[3] Jones JL (2014) *Toxoplasma gondii* seroprevalence in the United States 2009–2010 and comparison with the past two decades. American Journal of Tropical Medicine & Hygiene 90, 1135–1139. 10.4269/ajtmh.14-0013 PMC4047742 24710615

[4] Minor T (2015) The per case and total annual costs of foodborne illness in the United States. Risk Analysis 35, 1125–1139. 25557397 10.1111/risa.12316

[5] Scallan E (2011) Foodborne illness acquired in the United States – major pathogens. Emerging Infectious Diseases 17, 7–15. 21192848 10.3201/eid1701.P11101 PMC3375761

[6] Dubey JP, Lindsay DS and Speer CA (1998) Structures of *Toxoplasma gondii* tachyzoites, bradyzoites, and sporozoites and biology and development of tissue cysts. Clinical Microbiology Reviews 11, 267–299. 9564564 10.1128/cmr.11.2.267 PMC106833

[7] Hill DE, Chirukandoth S and Dubey JP (2005) Biology and epidemiology of *Toxoplasma gondii* in man and animals. Animal Health Research Reviews 6, 41–61. 16164008 10.1079/ahr2005100

[8] Montoya JG and Liesenfeld O (2004) Toxoplasmosis. Lancet (London, England) 363, 1965–1976. 10.1016/S0140-6736(04)16412-X 15194258

[9] Montoya JG, Boothroyd JC and Kovacs JA (2015) *Toxoplasma gondii*. In Bennett JE, Dolin R, Blaser MJ (eds), Principles and Practice of Infectious Diseases, 8th Edn. Philadelphia, PA: Elsevier Saunderss, pp. 3123–3151.

[10] Derouin F and Pelloux H (2008) Parasitology ESGoC. Prevention of toxoplasmosis in transplant patients. Clinical Microbiology & Infection 14, 1089–1101. 19018809 10.1111/j.1469-0691.2008.02091.x

[11] Robert-Gangneux F and Darde ML (2012) Epidemiology of and diagnostic strategies for toxoplasmosis. Clinical Microbiology Reviews 25, 264–296. 22491772 10.1128/CMR.05013-11 PMC3346298

[12] Dubey JP and Jones JL (2008) *Toxoplasma gondii* infection in humans and animals in the United States. International Journal of Parasitology 38, 1257–1278. 18508057 10.1016/j.ijpara.2008.03.007

[13] Sutterland AL (2015) Beyond the association. *Toxoplasma gondii* in schizophrenia, bipolar disorder, and addiction: systematic review and meta-analysis. Acta Psychiatrica Scandinavica 132, 161–179. 25877655 10.1111/acps.12423

[14] Torrey EF, Bartko JJ and Yolken RH (2012) *Toxoplasma gondii* and other risk factors for schizophrenia: an update. Schizophrenia Bulletin 38, 642–647. 22446566 10.1093/schbul/sbs043 PMC3329973

[15] Torrey EF (2007) Antibodies to *Toxoplasma gondii* in patients with schizophrenia: a meta-analysis. Schizophrenia Bulletin 33, 729–736. 17085743 10.1093/schbul/sbl050 PMC2526143

[16] Arling TA (2009) *Toxoplasma gondii* antibody titers and history of suicide attempts in patients with recurrent mood disorders. Journal of Nervous & Mental Disease 197, 905–908. 10.1097/NMD.0b013e3181c29a23 20010026

[17] Pedersen MG (2012) *Toxoplasma gondii* infection and self-directed violence in mothers. Archives of General Psychiatry 69, 1123–1130. 22752117 10.1001/archgenpsychiatry.2012.668

[18] Said B (2017) Risk factors for acute toxoplasmosis in England and Wales. Epidemiology & Infection 145, 23–29. 27677229 10.1017/S0950268816002235 PMC9507381

[19] Bamba S (2017) Seroprevalence and risk factors of *Toxoplasma gondii* infection in pregnant women from Bobo Dioulasso, Burkina Faso. BMC Infectious Diseases 17, 482. 28693432 10.1186/s12879-017-2583-6 PMC5504642

[20] Alvarado-Esquivel C (2014) Seroprevalence of *Toxoplasma gondii* infection and associated risk factors in Huicholes in Mexico. Parasites & Vectors 7. 10.1186/1756-3305-7-301 PMC4226977 24984845

[21] Nash JQ (2005) Risk factors for toxoplasmosis in pregnant women in Kent, United Kingdom. Epidemiology & Infection 133, 475–483. 15962554 10.1017/s0950268804003620 PMC2870271

[22] Jones JL (2009) Risk factors for *Toxoplasma gondii* infection in the United States. Clinical Infectious Diseases 49, 878–884. 19663709 10.1086/605433

[23] Iddawela D, Vithana SMP and Ratnayake C (2017) Seroprevalence of toxoplasmosis and risk factors of *Toxoplasma gondii* infection among pregnant women in Sri Lanka: a cross sectional study. BMC Public Health 17, 930. 29202747 10.1186/s12889-017-4941-0 PMC5716377

[24] Retmanasari A (2017) Prevalence and risk factors for toxoplasmosis in Middle Java, Indonesia. Ecohealth 14, 162–170. 27830388 10.1007/s10393-016-1198-5 PMC5357302

[25] Wam EC (2016) Seroprevalence of *Toxoplasma gondii* IgG and IgM antibodies and associated risk factors in women of child-bearing age in Njinikom, NW Cameroon. BMC Research Notes 9, 406. 27528009 10.1186/s13104-016-2206-0 PMC4986271

[26] Zhang XX (2016) Seroprevalence and associated risk factors of *Toxoplasma gondii* infection in the Korean, Manchu, Mongol and Han ethnic groups in eastern and northeastern China. Epidemiology & Infection 144, 2018–2024. 26833424 10.1017/S0950268815003337 PMC9150644

[27] Messier V (2009) Seroprevalence of *Toxoplasma gondii* among Nunavik Inuit (Canada). Zoonoses & Public Health 56, 188–197. 18811673 10.1111/j.1863-2378.2008.01177.x

[28] Meier HC (2016) Early life socioeconomic position and immune response to persistent infections among elderly Latinos. Social Sciences & Medicine 166, 77–85. 10.1016/j.socscimed.2016.07.004 PMC5573138 27543684

[29] Hill D (2011) Identification of a sporozoite-specific antigen from *Toxoplasma gondii*. Journal of Parasitology 97, 328–337. 10.1645/GE-2782.1 PMC3684278 21506817

[30] Kraybill D, Johnson-Weiner J and Nolt S (2013) The Amish. Baltimore, Maryland. USA: The John Hopkins University Press.

[31] Cuyun Carter GB (2011) Dietary intake, food processing, and cooking methods among Amish and non-Amish adults living in Ohio Appalachia: relevance to nutritional risk factors for cancer. Nutrition & Cancer 63, 1208–1217. 22026912 10.1080/01635581.2011.607547 PMC3800012

[32] Duffy AR (2019) *Toxoplasma gondii* serointensity and seropositivity: heritability and household-related associations in the Old Order Amish. International Journal of Environmental Research and Public Health 16. 10.3390/ijerph16193732 PMC6801611 31623376

[33] McDonald JH (2014) Handbook of Biological Statistics, 3rd Edn. Baltimore, Maryland: Sparky House Publishing, pp. 59–67.

[34] Almasy L and Blangero J (2010) Variance component methods for analysis of complex phenotypes. Cold Spring Harbor Protocols 2010, pdb top77. 10.1101/pdb.top77 PMC3064490 20439422

[35] Alvarado-Esquivel C (2010) Seroepidemiology of *Toxoplasma gondii* infection in a Mennonite community in Durango State, Mexico. Journal of Parasitology 96, 941–945. 10.1645/GE-2477.1 20481662

[36] Alvarado-Esquivel C (2011) Seroepidemiology of *Toxoplasma gondii* infection in general population in a northern Mexican city. Journal of Parasitology 97, 40–43. 10.1645/GE-2612.1 21348604

[37] Wilking H (2016) Prevalence, incidence estimations, and risk factors of *Toxoplasma gondii* infection in Germany: a representative, cross-sectional, serological study. Scientific Reports 6, 22551. 26936108 10.1038/srep22551 PMC4776094

[38] Taylor MR (1997) Community study of toxoplasma antibodies in urban and rural schoolchildren aged 4 to 18 years. Archives of Disease in Childhood 77, 406–409. 9487962 10.1136/adc.77.5.406 PMC1717368

[39] Muñoz-Zanzi C, Williams-Nguyen J and Belongia EA (2013) A sero-survey of toxoplasmosis in farm and non-farm children from Wisconsin, United States, 1997–1999. BMC Public Health 13, 837. 24025220 10.1186/1471-2458-13-837 PMC3847651

[40] Jones JL (2001) *Toxoplasma gondii* infection in the United States: seroprevalence and risk factors. American Journal of Epidemiology 154, 357–365. 11495859 10.1093/aje/154.4.357

[41] Costard S (2017) Outbreak-related disease burden associated with consumption of unpasteurized cow's milk and cheese, United States, 2009–2014. Emerging Infectious Diseases 23, 957–964. 28518026 10.3201/eid2306.151603 PMC5443421

[42] Boughattas S (2015) Commentary on: ‘detection of *Toxoplasma gondii* in raw caprine, ovine, buffalo, bovine, and camel milk using cell cultivation, cat bioassay, capture ELISA, and PCR methods in Iran’. Frontiers in Microbiology 6, 215. 25852672 10.3389/fmicb.2015.00215 PMC4364284

